# Supplementary material for: Effects of core stability exercises on balance among Chinese children and youth with intellectual disabilities
Source: PeerJ. 2025 Dec 9;13:e20454. doi: 10.7717/peerj.20454 (PMC12700112; doi:10.7717/peerj.20454)
Supplement: Supplemental Information 3 [file peerj-13-20454-s003.doc]

**核心稳定性训练对智力障碍儿童青少年平衡能力的影响**

**研究计划书**

目录

[1 研究假设及研究目的 3](#__RefHeading___Toc192442386)

[1.1 研究假设 3](#__RefHeading___Toc192442387)

[1.2 研究目的 3](#__RefHeading___Toc192442388)

[2 实验对象 3](#__RefHeading___Toc192442389)

[2.1 实验对象纳入标准 3](#__RefHeading___Toc192442390)

[2.2 实验对象排除标准 3](#__RefHeading___Toc192442391)

[2.3 实验对象脱落标准 3](#__RefHeading___Toc192442392)

[3 实验设计 4](#__RefHeading___Toc192442393)

[4 实验内容 4](#__RefHeading___Toc192442394)

[5 评估方案 6](#__RefHeading___Toc192442395)

[5.1 人口统计学信息 6](#__RefHeading___Toc192442396)

[5.2 静态平衡能力测试 6](#__RefHeading___Toc192442397)

[5.3 动态平衡能力测试 6](#__RefHeading___Toc192442398)

[6 实验注意事项 7](#__RefHeading___Toc192442399)

# 1 研究假设及研究目的

## 1.1 研究假设

核心稳定性训练可以有效提高智力障碍儿童青少年的平衡能力。

## 1.2 研究目的

探讨核心稳定性训练对智力障碍儿童青少年平衡能力的影响。

# 2 实验对象

结合研究需要和现实条件，选取38名温州市特殊教育学校6-18岁智力障碍学生为实验对象。实验前，所有家长及监护人需知情并签署知情同意书。本研究样本量的确定有两方面依据。一方面，为了精确的计算样本量，使用G*power软件中的T检验，效应量选择0.5，检验标准α = 0.05，检验效能1-β = 0.8，10%的脱落率，计算得出样本量为38人。另一方面，参照以往6项有关于核心稳定性训练对智力障碍儿童青少年平衡能力影响的研究，样本量在16-45人，本研究的被试量与这些研究相当。

## 2.1 实验对象纳入标准

（1）符合《精神疾病诊断与统计手册》第5版（DSM-5）中智力障碍诊断标准；

（2）年龄在6-18岁的男生和女生；

（3）能够听从指令或执行干预相关命令。

（4）除参加学校常规体育活动外，没有参加长期规律的核心稳定性和平衡相关训练计划。

## 2.2 实验对象排除标准

（1）有继发性身体疾病，如患有肢体残疾、视力残疾、听力残疾、脑瘫；

（2）患有任何神经系统疾病，如癫痫等；

（3）患有严重的心脑血管疾病，如高血压、心脏病等；

## 2.3 实验对象脱落标准

（1）实验期间不愿意继续进行，主动退出者；

（2）缺席超过4次以上或连续2次训练。

# 3 实验设计

1. 本研究采用单盲随机对照实验设计，采用分层随机抽样的方法确保各组包含相似数量的性别和儿童青少年受试对象。
2. 采用随机数表进行随机化分组。

（3）采用信封法进行分配隐藏。准备38张纸条作为干预组（纸条写A；19张）与对照组（纸条写B；19张），将38个不透明信封从1-38进行编号，依据随机数表放入相应的纸条，并将信封分为四组，分别为儿童女、儿童男、青少年女、青少年男，依据随机数表将纸条每一名受试对象前测结束后去专门保管信封的研究人员处打开相应组别的信封（按每组前测结束顺序由小到大打开相应编号的信封，例如儿童女组第一个完成前测的学生打开儿童女组的1号信封，以此类推），该研究人员记录该名受试对象的分组。最后汇总四组抽到A、B纸条的学生，完成分组，并通知他们A组在干预时间进行干预。**注：该名研究人员不得参与前测，通知A组、B组学生及其家长和老师时不得采用“干预组/实验组”与“对照组”的表述。**

# 4 实验内容

实验组在学校常规学习和生活的基础上额外进行核心稳定性训练，对照组则保持学校常规学习和生活不进行额外的训练。核心稳定性训练方案见表1所示。

**表1 核**心稳定性训练方案

| 周数 | 训练内容 | 训练频率 |
| --- | --- | --- |
| 1-2 | 1、仰卧位时收缩腹肌 | 3组，每组20个 |
|  | 2、俯卧位时收缩腹肌 | 3组，每组20个 |
|  | 3、蹲下时收缩腹部肌肉 | 3组，每组20个 |
| 3 | 1、仰卧位时收缩腹肌，一条腿伸展，另一条腿膝盖弯曲，紧贴腹部 | 3组，每组20个 |
|  | 2、俯卧位时收缩腹部肌肉，一侧腿伸直，另一侧腿屈膝 | 3组，每组20个 |
|  | 3、身体两侧的侧桥 | 6组，每组10秒 |
| 4 | 1、仰卧位收缩腹部肌肉，四肢向上，手臂和腿靠近 | 3组，每组20个 |
|  | 2、蹲下姿势，抬起一条腿，向后拉 | 每条腿3组，每组20次 |
|  | 3、双手握住重物时躯干转体 | 每边3组，每组20次 |
| 5 | 1、坐在瑞士球上屈膝收腹 | 3组，每组10秒 |
|  | 2、手举瑞士球深蹲 | 3组，每组15个 |
|  | 3、俯卧时同时抬高腿和手臂 | 3组，每组15次 |
| 6 | 1、向左或向右弯曲45度 | 每边3组，每组12次 |
|  | 2、当肩膀和手在地板上，抬起一条腿 | 每条腿3组，每组15次 |
|  | 3、仰卧在瑞士球上时收缩腹部肌肉 | 3组，每组12次 |

# 5 评估方案

在干预前后收集智力障碍学生的人口统计学信息，并对其平衡能力的各项指标开展评估，以验证核心稳定性训练干预的效果。

## 5.1 人口统计学信息

社会人口学指标包括性别、年龄、身高、体重和智力障碍程度。除年龄和智力障碍程度需由学校提供外，其他信息由现场测量获得（见表2）。

**表2 智力障碍学生人口统计学信息**

| 测试指标 | 信息来源 |
| --- | --- |
| 年龄 | 由学校提供 |
| 障碍程度 | 由学校提供 |
| 身高 | 测量获得 |
| 体重 | 测量获得 |

## 5.2 静态平衡能力测试

使用单腿直立检查法（one leg stand test, OLST）评估受试者的静态平衡能力。受试者优势脚单脚站立（根据个人情况确定左脚或者右脚），双手叉腰，观察睁眼、闭眼保持平衡的时间，以秒为单位进行计算。睁眼测试时保持眼睛平视前方（测试时如有条件可以面向墙壁，在与受试者眼睛平行高度的墙壁上做一个标记或贴一个标志物，让受试者在测试时看标志物），时间越长，平衡能力越好。

当出现以下情况之一时测试结束：

（1）抬起的脚接触了地面；

（2）手臂用来保持平衡；

（3）闭眼条件下睁开了眼睛。

为调整动作和睁开或闭上眼睛提供10秒的休息间隔。

## 5.3 动态平衡能力测试

使用Y平衡测试（Y Balance Test, YBT）评估受试者的静态平衡能力。Y平衡测试分3个测试方向，受试者在测试时单腿站立姿势下脚尖朝向即为前方，并据此将3个测试方向确定为前侧、后内侧和后内侧。同时，在进行平衡测试之前，首先要测量下肢长度，具体方法为：测试者使用卷尺测量髂前上棘到同侧脚内踝远端的距离。

正式测试，以右脚支撑为例：

（1）受试者光脚进行该项测试（减少由鞋子提供的额外的平衡与稳定），右脚站在测试平台上，并与测试平台平行，拇趾对准测试平台上的红色起始线。同时，双手掐腰，以排除可能由上肢参与的平衡。

（2）受试者保持右脚支撑，将左脚（自由脚）尽可能远地推动测试板内侧面（靠近测试平台的一侧），为了减轻受试者的疲劳，提高数据的准确性，受试者需左脚在同一方向上连续测3次，然后换右脚在该方向上继续连续测3次（此时为左脚支撑），并按照前侧、后内侧、后外侧的顺序依次进行。测试板内侧面在测试杆上对应的刻度即为受试者左脚（或者右脚，以自由脚为准）在该方向上的测试结果，并由测试者记录，精确到cm。

受试者在某个方向上能够按照上述测试要求进行，并平稳的将脚收回到起始位置即算1次成功的测试，而出现以下几种情况均被视为无效的测试，需要在该方向上重新进行测试，直到总共完成三个有效的试验：

（1）在测试时，支撑脚的脚后跟抬起或者支撑脚发生移动；

（2）自由脚在向各个方向进行测试时，以测试板或测试杆作为支撑；

（3）自由脚在收回时，身体因失去平衡而使自由脚触及地面；

（4）自由脚在伸出过程中没有与测试板始终贴合，或者在伸出末端用力猛踢测试板，使之靠惯性向前滑动。

# 6 实验注意事项

（1）考虑到实验对象的特殊性，个体差异性大，在实验过程中加强对训练的监督，根据学生的训练反映及承受情况，及时调整训练强度；

（2）实验前、后的测试要严格按照规定的测试步骤、方法进行。

（3）实验的评估和实验数据的统计分析由独立的研究人员完成。
