# Supplementary material for: Effects of core stability exercises on balance among Chinese children and youth with intellectual disabilities
Source: PeerJ. 2025 Dec 9;13:e20454. doi: 10.7717/peerj.20454 (PMC12700112; doi:10.7717/peerj.20454)
Supplement: Supplemental Information 4 [file peerj-13-20454-s004.doc]

**Effects of Core Stability Exercises on Balance in Chinese Children and Youth with Intellectual Disabilities**

**Trial protocol**

Directory

[1 Research hypothesis and research purpose 3](#__RefHeading___Toc192442195)

[1.1 Research hypothesis 3](#__RefHeading___Toc192442196)

[1.2 Research purpose 3](#__RefHeading___Toc192442197)

[2 Experimental subjects 3](#__RefHeading___Toc192442198)

[2.1 Inclusion criteria of experimental subjects 3](#__RefHeading___Toc192442199)

[2.2 Exclusion criteria of experimental subjects 3](#__RefHeading___Toc192442200)

[2.3 Experimental objects fall off standard 3](#__RefHeading___Toc192442201)

[3 Experimental design 4](#__RefHeading___Toc192442202)

[4 Experimental content 4](#__RefHeading___Toc192442203)

[5 Measurement of outcomes 6](#__RefHeading___Toc192442204)

[5.1 Assessment of anthropometry 6](#__RefHeading___Toc192442205)

[5.2 Assessment of static balance performance 6](#__RefHeading___Toc192442206)

[5.3 Assessment of dynamic balance performance. 6](#__RefHeading___Toc192442207)

[6 Experimental considerations 7](#__RefHeading___Toc192442208)

# 1 Research hypothesis and research purpose

## 1.1 Research hypothesis

Core stability exercises may improve the ability of children and youth with intellectual disability to maintain balance.

## 1.2 Research purpose

To explore the effects of core stability exercises on balance in Chinese children and youth with intellectual disabilities

# 2 Experimental subjects

Combined with the research needs and realistic conditions, 38 students with intellectual disability aged 6-18 in Wenzhou special education school were selected as experimental subjects. Before the experiment, all parents and guardians need to know and sign informed consent. The determination of the sample size of this study is based on two aspects. On the one hand, in order to accurately calculate the sample size, using the T test in G * power software, the effect size is 0.5, the test standard α = 0.05, the test efficiency 1-β = 0.8, and the 10 % shedding rate, the sample size is calculated to be 38 people. On the other hand, with reference to the previous 6 studies on the effect of core stability training on the balance ability of children and adolescents with intellectual disability, the sample size is 16-45, and the subjects of this study are equivalent to those of these studies.

## 2.1 Inclusion criteria of experimental subjects

(1) intellectual disability diagnosis based on the fifth edition of Diagnostic and Statistical Manual of Mental Disorder;

(2) aged 6–18 years;

(3) ability to follow instructions and communicate verbally;

(4) no history of core stability, strength and balance training in the past six months.

## 2.2 Exclusion criteria of experimental subjects

(1) uncontrolled cardiovascular disorder;

(2) neurological disorders (e.g. epilepsy);

(3) problems in vision or hearing that hinder exercise performance;

(4) musculoskeletal limitations to exercise, such as spinal, hip, knee or foot deformities;

## 2.3 Experimental objects fall off standard

(1) withdrawal from the study for any reason;

(2) Absent more than 4 times or 2 consecutive training.

# 3 Experimental design

(1) In this study, a single-blind randomized controlled trial design was used to ensure that each group contained a similar number of genders and children and adolescents by stratified random sampling.

(2) A random number table was used for randomization

(3) The envelopeis used for distribution concealment. Thirty-eight slips of paper were prepared as the intervention group (slips of paper were written A; 19 sheets) with the control group (note written B; 19 sheets), number 38 opaque envelopes from 1 to 38, put the corresponding paper strips according to the random number table, and divide the envelopes into four groups, namely children and girls, children and boys, adolescent girls, and adolescent boys, according to the random number table, each subject of the paper will go to the researcher who specially keeps the envelopes to open the envelopes of the corresponding group after the pretest is completed (open the corresponding numbered envelopes in the order of the end of the pretest in each group from small to large, for example, the first student in the children's female group to complete the pretest opens the No. 1 envelope of the children's female group, and so on), the researcher records the grouping of the subject. Finally, the four groups of students who drew A and B slips were summarized, the grouping was completed, and they were notified to intervene in group A at the intervention time. Note: The researcher is not allowed to participate in the pre-test, and the expressions "intervention group/experimental group" and "control group" should not be used when notifying the students in group A and group B, as well as their parents and teachers.

# 4 Experimental content

The experimental group underwent additional core stability training on the basis of the regular learning and life in school, while the control group maintained the regular learning and living in the school without additional training. The core stability training program is shown in Table 1.

Table 1 Jeffrey’s core stability exercises

| **Week** | **Form of exercise** | **The volume and intensity of exercise** |
| --- | --- | --- |
| 1 and 2 | Contracting abdominal muscles while lying in a supine position | Three sets and 20 repetitions in each set |
|  | Contracting abdominal muscles while lying in a prone position | Three sets and 20 repetitions in each set |
|  | Contracting abdominal muscles while in a squat position | Three sets and 20 repetitions in each set |
| 3 | Contracting abdominal muscles while lying in a supine position with one leg stretched and the other bent at the knee and pressed against the abdomen | Three sets and 20 repetitions in each set |
|  | Contracting abdominal muscles while lying in a prone position with one leg stretched and the body weight on the other leg which is bent at the knee | Three sets and 20 repetitions in each set |
|  | Side lying bridge for each side of the body | Six repetitions, a 10-s pause |
| 4 | Contracting abdominal muscles while lying in a supine position and pulling the limbs upward with arms and legs kept close | Three sets and 20 repetitions in each set |
|  | In squat position, one leg is raised and pulled outward and backward | Three sets for each leg and 20 repetitions in each set |
|  | Trunk rotation while holding weights in each hand | Three sets each part of the body and 20 repetitions in each set |
| 5 | Sitting on a Swiss ball and holding the abdomen in | Three sets, 10 s |
|  | Squatting while the Swiss ball is on the shoulder | Three sets and 15 repetitions for each set |
|  | Bringing up the arms and legs simultaneously in the prone position | Three sets and 15 repetitions for each set |
| 6 | Bending 45° to the left or right | Three sets for each side, 12 repetitions in each set |
|  | Bridging while shoulders and hands are on the floor and one leg is raised | Three sets for each leg, 15 repetitions in each set |
|  | Contracting abdominal muscles while lying in a supine position on the Swiss ball | Three sets, 12 repetitions in each set |

# 5 Measurement of outcomes

## 5.1 Assessment of anthropometry

Sociodemographic indicators include gender, age, height, weight, and intellectual disability severity. Except for age and degree of intellectual disability, which are to be provided by the school, other information is obtained from on-site measurements (see Table 2).

Body height (cm) and weight (kg) were measured using a portable instrument (Inbody BSM370, Korea). Height was measured to the nearest 0.10 cm in bare feet, whereas weight was examined to the nearest 0.10 kg. Then, body mass index (BMI; kg/m^2^) was calculated.

Table 2 Measure of Demographic Information of Students with Intellectual Disabilities

| Parameter | Sources of information: |
| --- | --- |
| Age | Provided by the school |
| Intellectual disability severity | Provided by the school |
| Height, m | Measured |
| Weight, kg | Measured |

## 5.2 Assessment of static balance performance

The one-legged stance (OLS) test was used in measuring the participants’ static balance performance. The participants were instructed to stand on their non-dominant leg, hands placed on hips, and then asked to perform the OLS as long as possible for 60 s. The OLS test has been used in children and youth, including those with intellectual disability (Rahmat & Hasan, 2013; Yılmaz et al. 2009).The assessment was conducted under four conditions: (1) standing with eyes opened on firm ground (EO, FI); (2) standing with eyes closed on firm ground (EC, FI); (3) standing with eyes opened on foam (i.e. AIREX Balance Pad) ground (EO, FO); (4) standing with eyes closed on foam ground (EC, FO). A trial was classified as invalid if the participants lost their balance (i.e. stepped with the lifted leg on the ground) or opened their eyes during the eyes closed conditions.

## 5.3 Assessment of dynamic balance performance.

The Lower Quarter Y Balance Test (YBT-LQ) Kit (Functional Movement Systems, Chatham, USA) was used to measure the participants’ dynamic balance performance. The test is a valid and reliable measure of dynamic balance in children and youth with intellectual disability (Ahmadi, Hasan & Hosin, 2012; Balayi, Sedaghati & Ahmadabadi, 2022; Jouira, Rebai & Sahli, 2023). The test kit consisted of a centralised stance platform to which three pipes attached represent the anterior (AT), posteromedial (PM) and posterolateral (PL) reach directions. Each pipe was marked in 1.0 cm increments for measurement purposes and was equipped with a moveable reach indicator.

Each participant was asked during the YBT-LQ to stand barefoot (to eliminate potential effects of varying footwear). One foot was on the center foot plate, and the most distal aspect of the toes was behind the starting line (Gribble, Hertel & Plisky, 2012). While maintaining a single-leg stance, each participant was asked to push the reach indicator with the reaching foot as far as possible and return to the original start position while maintaining balance. The test direction order was standardised as follows: right AT, right PM, right PL, left AT, left PM and left PL (Jouira, Rebai & Sahli, 2023). A trial was classified as invalid when the participants (1) lost their balance (i.e. stepped with the reach leg on the ground), (2) lifted the stance leg from the stance platform, (3) stepped on top of the reach indicator for support or (4) kicked the reach indicator (Muehlbauer & Waldermann, 2022; Plisky et al. 2009). When an invalid trial occurred, the respective data was discarded, and trials were repeated until a total of three valid trials were completed. The maximal reach distance (cm) per reach direction was used for further analysis.

# 6 Experimental considerations

(1) Considering the particularity of the experimental subjects and the large individual differences, the supervision of the training should be strengthened during the experiment, and the training intensity should be adjusted in time according to the students' training feedback and tolerance.

(2) The pre- and post-test tests should be carried out in strict accordance with the specified test procedures and methods.

(3) The evaluation of the experiment and the statistical analysis of the experimental data were done by independent researchers.
